# Supplementary material for: Activation of brown adipose tissue by a low-protein diet ameliorates hyperglycemia in a diabetic lipodystrophy mouse model
Source: Sci Rep. 2023 Jul 21;13:11808. doi: 10.1038/s41598-023-37482-6 (PMC10362023; doi:10.1038/s41598-023-37482-6)
Supplement: Supplementary file 2 — Supplementary Information 2. [file 41598_2023_37482_MOESM2_ESM.pdf]

Teklad Custom Diet

TD.140918

Amino Acid Diet (5% Protein, Orange)

Formula	g/Kg
L-Alanine	2.18
L-Arginine	1.46
L-Asparagine	4.79
L-Aspartic Acid	4.79
L-Cystine	1.67
L-Glutamic Acid	6.74
L-Glutamine	7.87
Glycine	0.69
L-Histidine HCl, monohydrate	1.07
L-Isoleucine	1.81
L-Leucine	5.9
L-Lysine HCl	4.74
L-Methionine	1.56
L-Phenylalanine	1.54
L-Proline	1.72
L-Serine	1.72
L-Threonine	2.26
L-Tryptophan	0.79
L-Tyrosine	1.61
L-Valine	1.95
Sucrose	291.248
Corn Starch	243.79
Maltodextrin	243.79
Corn Oil	52.0
Olive Oil	29.0
Cellulose	30.0
Mineral Mix, AIN-93M-MX (94049)	35.0
Calcium Phosphate, monobasic, monohydrate	8.2
Vitamin Mix, Teklad (40060)	10.0
TBHQ, antioxidant	0.012
Orange Food Color	0.1

Footnote

An amino acid defined diet with a calculate protein level of 5%. An orange food dye is added. Part of the series of amino acid adjusted diets (TD.140711 - TD.140712 & TD.140918 - 140919).

Key Features

+ **Amino Acid Defined Diet**
+ **Rodent Diet**
+ **Color Coded Orange**

Selected Nutrient Information[1]

	% by weight	% kcal from
**Protein**	5.0	5.1
**CHO**	75.4	76.4
**Fat**	8.1	18.5
**Kcal/g**	**3.9**	

[1] Calculated values

[2] Protein based on N x 6.25

Teklad Diets are designed & manufactured for research purposes only.

Key Planning Information

+ *Products are made fresh to order*
+ Store product at 4°C or lower
+ Use within 6 months (applicable to most diets)
+ Box labeled with product name, manufacturing date, and lot number
+ Replace diet at minimum once per week
  More frequent replacement may be advised
+ Lead time:
  · 2 weeks non-irradiated
  · 4 weeks irradiated

Product Specific Information

+ 1/2" Pellet or Powder (free flowing)
+ Minimum order 3 Kg
+ Irradiation available upon request

Options (Fees Will Apply)

+ Rush order (pending availability)
+ Irradiation (see Product Specific Information)
+ Vacuum packaging (1 and 2 Kg)

Speak With A Nutritionist

+ **(800) 483·5523**
+ **askanutritionist@envigo.com**

Contact Us

Obtain Pricing · Check Order Status

+ **teklad@envigo.com**
+ **(800) 483·5523**

International Inquiry *(Outside USA or Canada)*

+ **askanutritionist@envigo.com**

Place Your Order (USA & Canada)

Please Choose One

+ **www.envigo.com/teklad-orders**
+ **tekladorders@envigo.com**
+ **(800) 483·5523**
+ **(608) 277·2066** *facsimile*

TMH

© 2015 Envigo

04/25/17
